# Prediction model of emergency mortality risk in patients with acute upper gastrointestinal bleeding: a retrospective study

**DOI:** 10.7717/peerj.11656

**Published:** 2021-06-24

**Authors:** Lan Chen, Han Zheng, Saibin Wang

**Affiliations:** 1 Nursing Education Department, Affiliated Jinhua Hospital, Zhejiang University School of Medicine, Jinhua Municipal Central Hospital, Jinhua, ZheJiang, China; 2 Emergency Department, Affiliated Jinhua Hospital, Zhejiang University School of Medicine, Jinhua Municipal Central Hospital, Jinhua, ZheJiang, China; 3 Department of Respiratory Medicine, Affiliated Jinhua Hospital, Zhejiang University School of Medicine, Jinhua Municipal Central Hospital, Jinhua, ZheJiang, China

**Keywords:** Acute upper gastrointestinal bleeding, Prognosis, Nomogram, Emergency care, Mortality risk

## Abstract

**Background:**

Upper gastrointestinal bleeding is a common presentation in emergency departments and carries significant morbidity worldwide. It is paramount that treating physicians have access to tools that can effectively evaluate the patient risk, allowing quick and effective treatments to ultimately improve their prognosis. This study aims to establish a mortality risk assessment model for patients with acute upper gastrointestinal bleeding at an emergency department.

**Methods:**

A total of 991 patients presenting with acute upper gastrointestinal bleeding between July 2016 and June 2019 were enrolled in this retrospective single-center cohort study. Patient demographics, parameters assessed at admission, laboratory test, and clinical interventions were extracted. We used the least absolute shrinkage and selection operator regression to identify predictors for establishing a nomogram for death in the emergency department or within 24 h after leaving the emergency department and a corresponding nomogram. The area under the curve of the model was calculated. A bootstrap resampling method was used to internal validation, and decision curve analysis was applied for evaluate the clinical utility of the model. We also compared our predictive model with other prognostic models, such as AIMS65, Glasgow-Blatchford bleeding score, modified Glasgow-Blatchford bleeding score, and Pre-Endoscopic Rockall Score.

**Results:**

Among 991 patients, 41 (4.14%) died in the emergency department or within 24 h after leaving the emergency department. Five non-zero coefficient variables (transfusion of plasma, D-dimer, albumin, potassium, age) were filtered by the least absolute shrinkage and selection operator regression analysis and used to establish a predictive model. The area under the curve for the model was 0.847 (95% confidence interval [0.794–0.900]), which is higher than that of previous models for mortality of patients with acute upper gastrointestinal bleeding. The decision curve analysis indicated the clinical usefulness of the model.

**Conclusions:**

The nomogram based on transfusion of plasma, D-dimer, albumin, potassium, and age effectively assessed the prognosis of patients with acute upper gastrointestinal bleeding presenting at the emergency department.

## Introduction

Acute upper gastrointestinal bleeding (AUGIB) is a common presentation at emergency departments (EDs), with hematemesis, melena, and syncope representing as the major symptoms. Although the survival rate of patients with AUGIB has improved with advances in drug therapy and endoscopic diagnosis and treatment, it remains a potentially life-threatening gastrointestinal emergency (Balaban et al., 2014). It is paramount that treating physicians have access to tools that can effectively evaluate the patient risk, allowing quick and effective treatments to ultimately improve their prognosis (Balaban et al., 2014; Barkun et al., 2019).

Several previous models have been developed to estimate the mortality risk of patients with UGIB, such as the Glasgow-Blatchford score (GBS), Rockall score (RS), and the AIMS65 score (Rockall et al., 1996; Blatchford, Murray & Blatchford, 2000; Saltzman et al., 2011; Gaduputi et al., 2014; Abougergi et al., 2016). However, existing predictive models are not commonly used in clinical care for various reasons. First, some of these models require endoscopic information that is not readily available at the time of presentation. However, given the availability of endoscopy, the use of either GBS, modified GBS (mGBS), Pre-Endoscopic RS (PERS), or AIMS65 score is currently recommended, as these models are based on data that are easy to acquire. For example, GBS includes the measurement of systolic blood pressure (SBP), hemoglobin (HB), blood urea nitrogen (BUN), albumin, and international normalized ratio (INR), pulse, as well as evaluation of the presence of symptoms such as melena, syncope and liver disease, and heart failure, while PERS considers age, state of shock, and concomitant disease. Second, recent studies have found inconsistent results when using these models (Yaka et al., 2015; Budimir et al., 2016; Martinez-Cara et al., 2016; Park et al., 2016; Stanley et al., 2017; Zhou et al., 2017; Tang et al., 2018; Kim, Choi & Shin, 2019; Robertson et al., 2020). Third, it is difficult to obtain a personalized mortality assessment from these systems. More recently, Zhou et al. developed a prognostic nomogram for cirrhosis patients with UGIB admitted to intensive care units that they named upper gastrointestinal bleeding-chronic liver failure-sequential organ failure assessment (UGIB-CLIF-SOFA) (Zhou et al., 2017). However, as cirrhosis accounts only for a subset of patients with UGIB, the use of this model is limited. Moreover, the type of clinical intervention administered is an important factor affecting patients’ prognosis. To the best of our knowledge, a prognostic model that includes patient characteristics and clinical interventions as risk factors affecting prognosis remains to be developed. In addition, no current prognostic model can predict individual level mortality probability for patients with AUGIB presenting at EDs.

Various factors influence the prognosis of patients with AUGIB, including primary disease, volume and site of bleeding, age and intervention administered at the time of admission (Lahiff et al., 2012; Ko et al., 2017; Barkun et al., 2019). Data on bleeding-related factors, such as details of the site, cause, or type of bleeding, are difficult to obtain for patients treated in emergency care. The extent of bleeding should be ascertained on the basis of the patient’s chief complaint, vital signs, and laboratory test results. The aim of this study was to develop a model to predict the mortality of individual patients presenting at EDs, based on routinely collected, objective parameters, and clinical interventions administered. The model is accompanied by a nomogram.

## Materials & methods

### Research design

This was a retrospective cohort study of consecutive patients with AUGIB treated at an ED between July 2016 and June 2019. The Ethics Committee of Jinhua Municipal Central Hospital approved this study (No.16–32). The data were anonymous, and the requirement for informed consent was therefore waived. The study adhered to the principles of the Declaration of Helsinki.

### Study population

The medical records were used to recognize participants. Patients older than 18 years of age who had hematemesis (or bloody nasogastric aspirate), melena, or both, as confirmed by the hospital staff, were considered for inclusion (Villanueva et al., 2013). Patients were excluded from the present study if they sought treatment for melena but are diagnosed with lower gastrointestinal bleeding. A total of 991 patients were included in this study.

### Data collection

Structured data extraction forms were used by trained researchers for data collection. Medical record review and data extraction were performed by nursing staff with >10 years of experience at the ED. Following data extraction, quality control was performed on randomly selected data subsets. The following data were extracted: age (years), gender, chief complaints (syncope, hematemesis, melena), first effective vital signs and pulse oxygen saturation (%; in room air) obtained at the time of emergency admission, laboratory tests of blood, comorbidities, type of blood transfusion (mL), endoscopic reports, and survival status at discharge from ED (death in ED or within 24 h after leaving the ED). AIMS65, GBS, mGBS, and PERS were calculated for every patient based on the first available data. At the study site, the first vital signs are recorded in the emergency triage system, which constitutes part of the electronic medical records. Data on blood test results and clinical interventions can also be accessed through electronic medical records.

### Statistical analysis

Data were analysised as previously described in Wang, Tu & Dong (2019), especially the process of model building and model validation. We used descriptive statistics to summarize the patients’ baseline characteristics. For categorical variables, counts (percentages) were reported, while for continuous variables, mean ± standard deviation (SD) or median (interquartile) were reported. Comparisons of parameters between survivors and non-survivors were performed using Student’s t-test and the Mann–Whitney U test for continuous variables with or without a normal distribution and using the χ^2^-test for categorical variables. The least absolute shrinkage and selection operator (LASSO) regression, which is suitable for the regression of high-dimensional data (Huang et al., 2016), was used for predictor selection and regularization. Logistic regression analysis was used to establish a model predicting death in ED or within 24 h after leaving the ED, and a nomogram was constructed on the basis of this model. The area under the curve (AUC) was calculated to evaluate the discrimination performance of the model and a 1,000 bootstrap method was applied for internal validation. For further validation, enrolled patients were randomly divided into two groups, 50% for training and 50% for validating. Statistical differences in the AUCs were compared using the method of DeLong. Decision curve analysis (DCA) was performed to evaluate the model’s clinical utility by quantifying the net benefits at different threshold probabilities (Vickers et al., 2008). Furthermore, we calculated the AUC and DCA to evaluate the predictive performance of the nomogram in undergoing and not undergoing endoscopy subgroup. The predictive performances of the nomogram, AIMS65, GBS, mGBS, and PERS were compared by AUC and net reclassification improvement (NRI). Statistical analysis was conducted using EmpowerStats (www.empowerstats.com) and R 3.5.1 (www.r-project.org). *P*-values < 0.05 were considered statistically significant.

## Results

**Figure 1:**
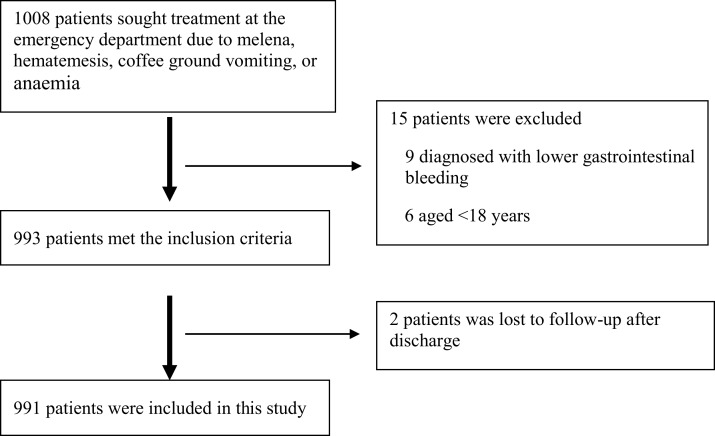
Patient cohort. The flow of patients included in the study is shown.

A total of 1,008 patients were initially recruited into the study and 991 remained after the exclusion criteria were applied (Fig. 1).

Table 1 presents the patient characteristics and types of clinical interventions administered. Among the 991 patients, 41 (4.14%) died in ED or within 24 h after leaving the ED. Univariate analysis showed that death in ED occurred more frequently in patients with low red blood cell (RBC) count, hematocrit (HCT), HB, platelet (PLT) count, albumin (ALB), albumin/globulin ratio (A/G), total protein, in patients with high C-reactive protein (CRP), prothrombin time (PT), D-dimer, INR, glutamic oxaloacetic transaminase (AST), serum creatinine, in patients with chronic liver diseases, in patients with lower pulse oxygen saturation and in patients receiving plasma transfusion.

**Table 1:** Baseline demographic and clinical characteristics of the study participants.


Variable	Death in emergency department		*P*
	No (*n* = 950)	Yes (*n* = 41)	
Baseline characteristics			
Sex, *n* (%)			0.119
Male	682 (71.8%)	34 (82.9%)	
Female	268 (28.2%)	7 (17.1%)	
Age (years)	64.6 ± 16.4	69.4 ± 13.4	0.065
Monitoring parameters at admission			
Temperature (degrees Celsius)	36.6 ± 0.8	36.2 ± 1.3	0.123
HR (beat/min)	94.5 ± 20.0	96.1 ± 27.6	0.620
SBP (mmHg)	120.0 ± 25.6	114.5 ± 34.9	0.179
DBP (mmHg)	66.1 ± 17.5	65.6 ± 30.2	0.095
Pulse oxygen saturation (%)	96.8 ± 5.9	89.1 ± 22.1	<0.001
Chief complaints, *n* (%)			
Syncope			0.198
No	913 (96.1%)	41 (100.0%)	
Yes	37 (3.9%)	0 (0.0%)	
Hematemesis			0.177
No	630 (66.3%)	23 (56.1%)	
Yes	320 (33.7%)	18 (43.9%)	
Melena			0.880
No	222 (23.4%)	10 (24.4%)	
Yes	728 (76.6%)	31 (75.6%)	
Blood test			
RBC (10^12^/L)	3.1 ± 1.0	2.5 ± 0.9	<0.001
HCT (%)	27.3 ± 9.0	23.2 ± 7.1	0.005
HB (g/L)	89.4 ± 32.9	74.4 ± 23.8	0.004
PLT (10^9^/L) Median (Q1–Q3)	173.0 (100.0–244.0)	106.0 (73.0–157.0)	0.001
CRP (mg/L) Median (Q1–Q3)	5.4 (2.0–20.4)	17.9 (3.0–44.7)	0.024
PT (s) Median (Q1–Q3)	13.0 (11.9–15.3)	18.1 (14.1–23.0)	0.007
D-dimer (μg/L) Median (Q1–Q3)	726.5 (320.0–2066.2)	3062.0 (1018.5–6258.5)	<0.001
INR Median (Q1–Q3)	1.1 (1.0–1.3)	1.6 (1.3–2.0)	<0.001
ALT (IU/L) Median (Q1–Q3)	18.9 (11.7–30.9)	28.5 (10.1–65.8)	0.105
AST (IU/L) Median (Q1–Q3)	26.6 (19.1–46.5)	46.4 (27.2–185.2)	<0.001
Blood amylase (U/L) Median (Q1–Q3)	58.0 (43.0–81.0)	55.5 (39.0–112.5)	0.992
Potassium (mmol/L)	4.1 ± 0.7	4.9 ± 1.3	<0.001
BUN (mg/dl)	11.8 ± 7.4	12.8 ± 7.3	0.428
Serum creatinine (μmol/L) Median (Q1–Q3)	96.0 (79.0–120.1)	140.5 (114.8–207.6)	<0.001
ALB (g/L)	31.6 ± 6.5	25.8 ± 5.8	<0.001
A/G	1.1 ± 0.3	1.0 ± 0.3	0.018
Total protein (g/L)	61.3 ± 10.6	53.7 ± 13.0	<0.001
Blood glucose (mmol/L) Median (Q1–Q3)	7.7 (6.5–10.0)	8.7 (6.3–11.1)	0.847
PH	7.4 ± 0.1	7.2 ± 0.3	<0.001
Lactic acid (mmol/L)	3.5 (1.2–4.6)	12.1 (3.4–18.5)	<0.001
Base excess (mmol/L)	−3.6 (−6.3–0.3)	−14.8 (−24.7–−3.4)	<0.001
Intervention measures			
Emergency observation time (h) Median (Q1–Q3)	19.0 (9.0–27.0)	14.0 (7.0–25.0)	0.460
Transfusion of red blood cells, *n* (%)			0.157
No	383 (40.3%)	12 (29.3%)	
Yes	567 (59.7%)	29 (70.7%)	
Transfusion of red blood cells (ml) Median (Q1–Q3)	2.9 (0.0–4.0)	3.7 (0.0–6.0)	0.066
Transfusion of plasma, *n* (%)			<0.001
No	642 (67.6%)	15 (36.6%)	
Yes	308 (32.4%)	26 (63.4%)	
Transfusion of plasma (ml) Median (Q1–Q3)	153.7 (0.0–200.0)	273.2 (0.0–400.0)	<0.001
Transfusion of cryoprecipitate, *n* (%)			0.590
No	899 (94.6%)	38 (92.7%)	
Yes	51 (5.4%)	3 (7.3%)	
Transfusion of platelets, *n* (%)			0.845
No	931 (98.0%)	40 (97.6%)	
Yes	19 (2.0%)	1 (2.4%)	
Comorbidities, *n* (%)			
Chronic liver diseases			0.017
No	787 (82.8%)	28 (68.3%)	
Yes	163 (17.2%)	13 (31.7%)	
Diabetes			0.969
No	882 (92.8%)	38 (92.7%)	
Yes	68 (7.2%)	3 (7.3%)	
Hypertension			0.829
No	823 (86.6%)	36 (87.8%)	
Yes	127 (13.4%)	5 (12.2%)	
Stroke			0.663
No	916 (96.4%)	39 (95.1%)	
Yes	34 (3.6%)	2 (4.9%)	
Hematological system diseases			0.449
No	923 (97.2%)	39 (95.1%)	
Yes	27 (2.8%)	2 (4.9%)	
Tumor			0.474
No	904 (95.2%)	38 (92.7%)	
Yes	46 (4.8%)	3 (7.3%)	
Heart failure, coronary heart disease			0.615
No	912 (96.0%)	40 (97.6%)	
Yes	38 (4.0%)	1 (2.4%)	
Respiratory failure			0.760
No	933 (98.2%)	40 (97.6%)	
Yes	17 (1.8%)	1 (2.4%)	
Kidney failure			0.478
No	904 (95.2%)	40 (97.6%)	
Yes	46 (4.8%)	1 (2.4%)	
Receive endoscopy			0.345
No	579 (60.9%)	28 (68.3%)	
Yes	371 (39.1%)	13 (31.7%)	
Source of bleeding according to endoscopic report			
Peptic ulcer	178 (48.0%)	7 (53.8%)	0.677
Gastric	52 (14.0%)	2 (15.4%)	0.889
Duodenal	117 (31.5%)	3 (23.1%)	0.518
Stomal	9 (2.4%)	2 (15.4%)	0.006
Gastroesophageal varices	110 (29.6%)	3 (23.1%)	0.609
Mallory–Weiss tears	11 (3.0%)	0 (0.0%)	0.529
Erosive gastritis or esophagitis	35 (9.4%)	0 (0.0%)	0.245
Neoplasms	13 (3.5%)	2 (15.4%)	0.030
Other	24 (6.5%)	1 (7.7%)	0.861

**Notes:**

**The definition of comorbidities.** Renal failure: previous history of kidney failure, on dialysis or GFR < 15 ml/min/1.73 m^2^_;_ Respiratory failure: PO_2_ < 60 mmHg or SO_2_ <90% on room air; Heart failure: current or past clinical symptoms (limitation of activity, fatigue and dyspnea or orthopnea), signs (edema, elevated jugular venous pressure, rales, or S3 gallop), or radiologic evidence of pulmonary congestion.

HR, heart rate; SBP, systolic blood pressure; DBP, diastolic blood pressure; RBC, red blood cell; HCT, hematocrit; HB, hemoglobin; PLT, platelet count; CRP, C-reactive protein; PT, prothrombin time; INR, international normalized ratio; ALT, alanine aminotransferase; AST, glutamic oxaloacetic transaminase; BUN, blood urea nitrogen; ALB, albumin; A/G, albumin /globulin ratio; GFR, glomerular filtration rate.

**Figure 2:**
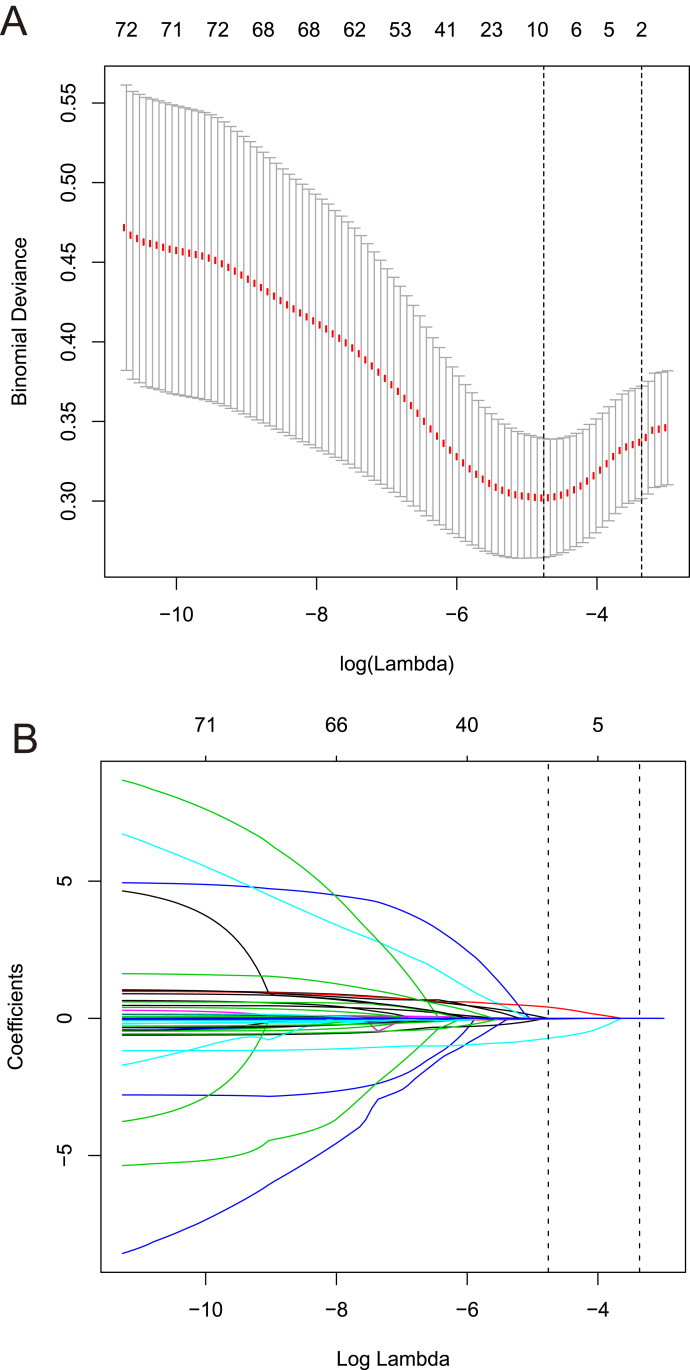
Predictors selection based on the least absolute shrinkage and selection operator (LASSO) regression method. (A) Tuning parameter (lambda) selection in the LASSO regression used 10-fold cross-validation. Binomial deviance was plotted versus log (lambda). The dotted vertical lines were drawn at the optimal values by using the 1-SE criteria. (B) LASSO regression coefficient profiles of variables. A coefficient profile plot was created against the log (lambda) sequence. Dotted vertical lines were drawn at the optimal values by using the 1-SE criteria. In the present study, predictors were chosen according to the 1-SE criteria, a total of five non-zero coefficients were filtered and used to construct the predictive model. SE, standard error.

**Figure 3:**
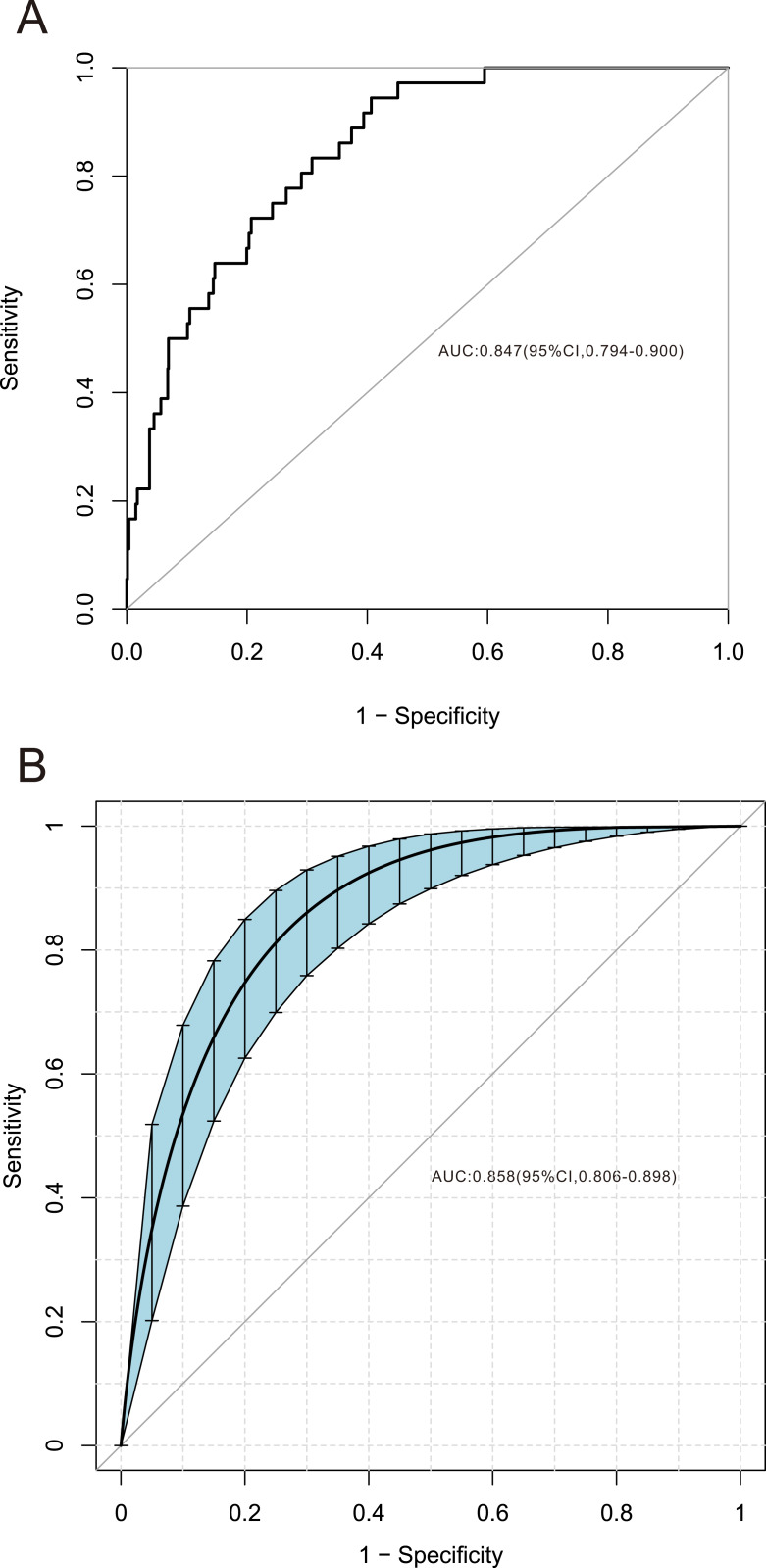
Receiver operating characteristic curve of the predictive model and in the internal validation model. The area under the curve (AUC) (A) shows the discrimination ability of the model, and AUC (B) of the internal validation model. The shaded blue portion represents the 95% confidence interval. CI, confidence interval.

**Figure 4:**
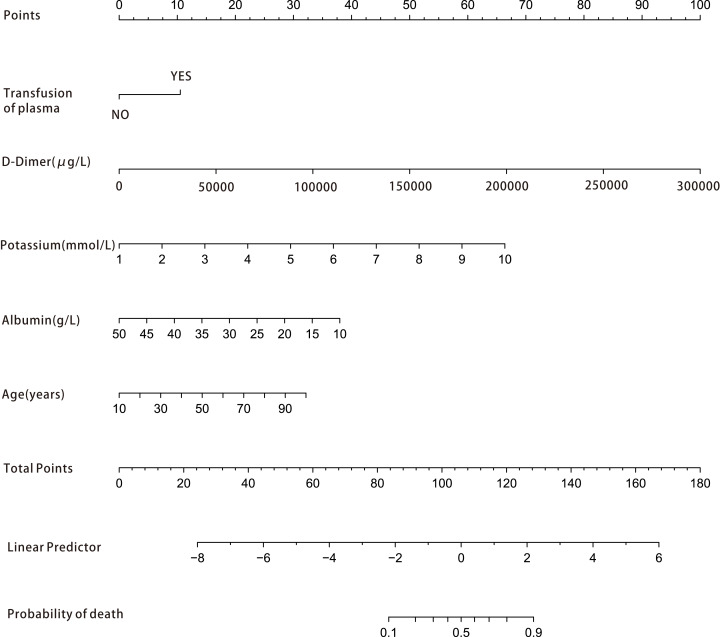
Nomogram for estimation of patient’s risk of death due to acute upper gastrointestinal bleeding (AUGIB) in the emergency department or within 24 h after leaving the emergency department. First, score each predictor value of an individual using the top scale. Second, sum up all the scores and identify the corresponding score on the scale. Finally, the corresponding risk of death in the emergency department or within 24 h after leaving the emergency department for the given patient is on the lowest rule. For example, a patient received transfusion of plasma in emergency department (10 point), D-dimer 5,000 μg/L (15 point), potassium 5 mmol/L (30 point), albumin 30 g/L (20 point) and 90 years old (30 point ), than the total score is 105 point and the risk of death is about 50%.

Among the 42 collected variables shown in Table 1, included age, gender, chief complaints, first effective vital signs and pulse oxygen saturation, laboratory tests of blood, comorbidities, type of blood transfusion, five were filtered on the basis of non-zero coefficients calculated by the LASSO regression analysis using the minimum criteria (Fig. 2). These variables were transfusion of plasma, D-dimer, albumin, potassium, and age. We constructed a mortality risk prediction model based on the aforementioned five predictors (Table 2). The AUC for the predictive model was 0.847 (95% confidence interval (CI) [0.794–0.900]), whereas the AUC for the internal validation model was 0.858 (95% CI [0.806–0.898]) (Fig. 3). The AUC for training group and validating group was 0.839 and 0.838, respectively (Fig. S1). The AUC of this model was also greater than that of the four other models (Table 3). We then calculated continuous NRI for the nomogram and four other models for death in ED or within 24 h after leaving the ED. The results also show that the nomogram was superior to other models (Table S1). The corresponding nomogram is presented in Fig. 4. The AUC for undergoing and not undergoing endoscopy subgroup was 0.874 and 0.822, respectively (Fig. S2).

**Table 2:** Logistic regression model and the Odds ratio of predictors.

Variable	*β*	OR (95% CI)	*P*
Transfusion of plasma, yes	1.163	2.913 [1.315–6.453]	0.008
ALB (g/L)	−0.089	0.918 [0.857–0.983]	0.014
Potassium (mmol/L)	0.779	2.020 [1.386–2.945]	0.000
Age (years)	0.036	1.037 [1.007–1.068]	0.015
D-dimer (μg/L)	0.001	1 [1–1.001]	0.003

**Notes:**

**Logistic regression model:** −7.335 + 1.163 × (Transfusion of plasma, yes) −0.089 × ALB (g/L) + 0.779 × Potassium (mmol/L) + 0.036 × Age (years) + 0.001 × D-dimer (μg/L).

ALB, albumin; CI, confidence interval; OR, odds ratio.

**Table 3:** AUC, sensitivity, specificity, positive predictive value, negative predictive value of different prognostic models to predict mortality risk in patients with AUGIB.

Models	Original AUC	95% CI	Sensitivity (%)	Specificity (%)	PPV (%)	NPV (%)	*P*^b^	Corrected AUC^a^	95% CI	*P*^b^
Nomogram	0.847	[0.794–0.900]	0.947	0.604	0.099	0.996		0.858	[0.806–0.898]	
GBS	0.647	[0.561–0.733]	0.650	0.632	0.076	0.974	<0.001	0.638	[0.542–0.715]	<0.001
MGBS	0.678	[0.599–0.757]	0.553	0.692	0.076	0.971	<0.001	0.693	[0.611–0.769]	<0.001
PERS	0.681	[0.606–0.756]	0.763	0.496	0.064	0.979	<0.001	0.696	[0.618–0.779]	<0.001
AIMS65	0.567	[0.483–0.651]	0.889	0.209	0.050	0.976	<0.001	0.573	[0.471–0.679]	<0.001

**Notes:**

a Using bootstrap 500.

b Compared with the predictive model that we developed.

AUGIB, acute upper gastrointestinal bleeding; GBS, Glasgow-Blatchford bleeding score; MGBS, modified Glasgow-Blatchford bleeding score; PERS, Pre-Endoscopic Rockall Score; AUC, area under the curve; CI, confidence interval; PPV, positive predictive value; NPV, negative predictive value.

**Figure 5:**
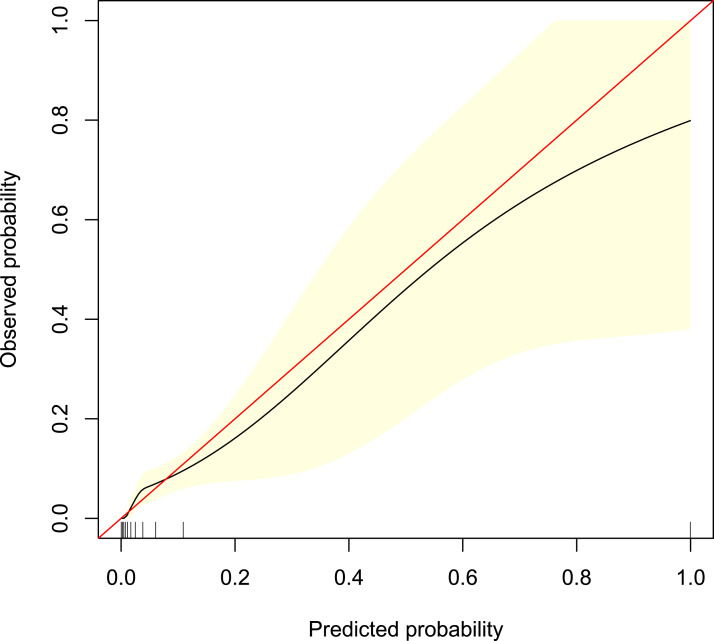
Calibration curve of the predictive model. It shows a good fit between the predicted risk of death and observed outcomes in patients with acute upper gastrointestinal bleeding. The red solid line represents an ideal predictive model, and the solid black line shows the actual performance of the predictive model. The yellow shadow represents a 95% confidence interval. The model overestimated mortality risk in patients with an predicted mortality great than 8%.

**Figure 6:**
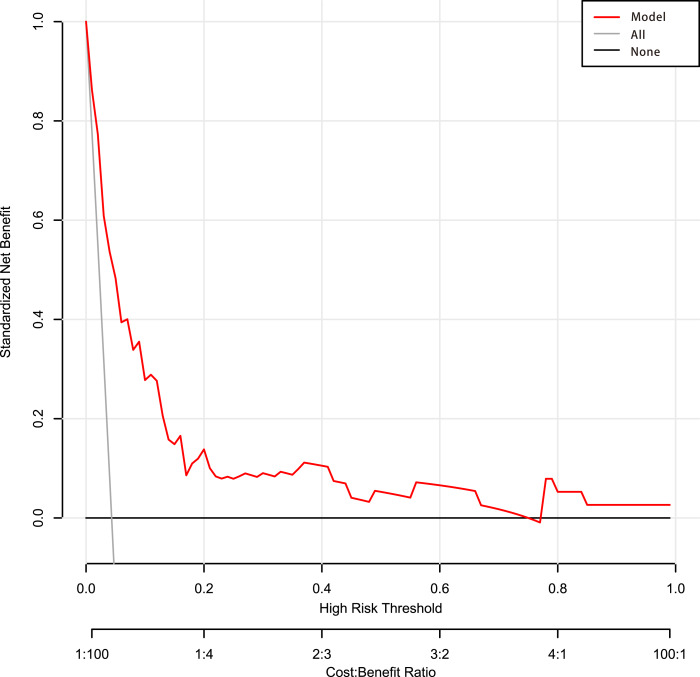
Decision curve analysis of the predictive model. Net benefit was produced against the high-risk threshold. The red solid line represents the predicted estimates. The decision curve indicates that when the threshold probability was within 3–76%, using this model in decision-making benefitted patient outcomes compared with either treat-all or treat-none strategies.

The calibration curve of the predictive model showed a good fit between the predicted risk of death and observed outcomes in patients with AUGIB (Fig. 5). The decision curve (Fig. 6) demonstrated that this model, by helping assess the risk of death in ED and informing interventions, improved patient outcomes compared with either treat-all or treat-none strategies. Specifically, when the model assessed the risk of death in ED as within 3–76%, the benefits of this model to patient outcomes were most robust.

## Discussion

This study proposed a novel and easy-to-use model for predicting death in ED or death within 24 h after leaving the ED of patients with AUGIB. This model combined five predictors selected by LASSO regression: transfusion of plasma, D-dimer, albumin, potassium, age. Based on patients’ characteristics and clinical interventions, all five predictors were readily accessible at EDs. Our nomogram may be used to infer the risk of mortality and therefore aid in the screening of patients with AUGIB who are at high risk. Thus, this could be a useful aid for physicians in determining a treatment regime. The model showed a good discriminatory ability of 0.847 (95% CI [0.794–0.900]). Moreover, it showed potential clinical usefulness. Although the calibration curve showed that among patients with actual mortality of great than 8%, the model overestimated mortality risk by 4% or greater, which may cause the waste of medical resources, it also would likely promote the active and efficient management of emergency patients.

The ability to quickly identify and appropriately treat patients at high risk is paramount in emergency care. Infusion of large plasma volumes was associated with poor survival outcomes (Subramaniam et al., 2016). Patients who received freshly frozen plasma transfusions had significantly greater rates of overall complications, acute respiratory distress syndrome, and pneumonia (Zhang et al., 2017), which resulted in a relatively higher mortality rate among these patients. D-dimer, as a fibrinolytic activity monitor, is generally used to evaluate the overall fibrinolytic condition in UGIB patients (Yue et al., 2020). Patients were considered to have hyperfibrinolysis if they concomitantly had high values of D-dimer and t-PA activity (Violl et al., 1996). Thus D-dimer is a stronger predictor for severity and prognosis both in Nonvariceal UGIB and variceal UGIB (Primignani et al., 2008; Yue et al., 2020).

Albumin has multiple biological characteristics, and hypoalbuminemia is a risk factor of mortality in certain diseases. The correlation between hypoalbuminemia and the prognosis of UGIB has also been reported (Lyles, Elliott & Rockey, 2014; Bunchorntavakul, Yodket & Singhasena, 2017). Many studies used the receiver operating characteristic (ROC) curve to find the best value of albumin for predicting mortality. Lower than 3.2 or 2.6 was used as criteria for hypoalbuminemia and were considered to be independent predictors of death (Gonzalez-Gonzalez et al., 2011, 2016; Chandnani et al., 2019). Our study showed that the lower the albumin, the greater the risk of death in the ED. With increasing age, the function of the human body gradually decreases, and comorbidities also increase. Therefore, advanced age is a risk factor for the prognosis of various diseases (Rajan et al., 2019). In the present study, the risk of death increases with age.

Interestingly, high serum potassium, which has never been clearly described as an outcome predictor of UGIB, was found to be an independent predictor for death in ED. Mortality risk progressively increased with dyskalemia in patients with and without heart failure, chronic kidney disease, and/or diabetes (Collins et al., 2017). Many studies have shown that serum potassium concentration correlates with the severity of cirrhosis, and mortality was also significantly higher in patients with hyperkalemia (Uslan, Olt & Eminler, 2015; Maiwall et al., 2016). Accordingly, it is likely that serum potassium can be another valuable predictor for death in patients presenting with UGIB.

There are many different scoring systems available to stratify patients with AUGIB at risk of rebleeding or death and to predict the need for clinical interventions such as transfusion and endoscopic therapy. GBS was established to identify patients at low or high risk of needing treatment (Blatchford, Murray & Blatchford, 2000), while RS and AIMS65 were developed to predict in-hospital mortality (Rockall et al., 1996; Saltzman et al., 2011). However, only some scores from these models are suitable for ED, and the results are inconsistent. The most widely studied scores are PERS, GBS, mGBS, and AIMS65 (Rout et al., 2019; Tham & Stanley, 2019). When compared with RS and PERS, GBS has been shown to be the best for predicting intervention or death (Park et al., 2016; Stanley et al., 2017). For predicting in-hospital mortality, other studies have shown that the area under the ROC curve (AUROC) of AIMS65 is superior to or similar to the GBS, RS, and PERS for patients with AUGIB (Yaka et al., 2015; Budimir et al., 2016; Martinez-Cara et al., 2016; Tang et al., 2018; Kim, Choi & Shin, 2019; Robertson et al., 2020). However, the predictive effects of these scoring systems for death ranged from 0.56 to 0.91 (Uslan, Olt & Eminler, 2015; Maiwall et al., 2016; Collins et al., 2017; Rajan et al., 2019; Rout et al., 2019; Tham & Stanley, 2019; Shung et al., 2020). The machine learning model developed by Shung et al., which aimed to identify patients meeting a composite endpoint of hospital-based intervention or death within 30 days, performed better than the GBS, admission-RS, and AIMS65 (Shung et al., 2020). However, an app is required in order to access this tool. In addition, most of these studies have focused on predicting hospital mortality or death within 30 days. An effective emergency death risk assessment model may be more valuable to emergency care providers. In our study, we included the parameters considered in the established scoring system and added other parameters that may be relevant to the prognosis, such as CRP, coagulation function tests, and laboratory test results that reflect the functions of important organs such as the liver and kidney (Makhlouf & Morsy, 2012; Tomizawa et al., 2014; Bunchorntavakul, Yodket & Singhasena, 2017). Also, we included transfusion of blood. Among the existing models, our results show that PERS is most effective for predicting in-ED mortality. Nevertheless, our new model outperformed PERS in both the original AUC and corrected AUC.

Nomograms are graphical depictions of individual-level prognosis predictions and have been shown to be more accurate than conventional models for predicting prognosis (Balachandran et al., 2015; Cooperman et al., 2016; Huang et al., 2016; Touijer & Scardino, 2009). We established a nomogram based on five predictors including four clinical patient characteristics, which can be readily obtained in clinical settings, and one medical intervention that can be used for the better management of patients. LASSO is a statistical formula mainly used for feature selection and regularization of data model. The LASSO method regularizes the parameters of the model by shrinking the regression coefficient and reduces some of them to zero. The feature selection phase occurs after the shrinkage, where each non-zero value is selected for use in the model (Chang et al., 2021). LASSO, which is considered superior to the conventional approach of choosing predictors, is commonly used in studies with a small sample size and a large number of predictors (Huang et al., 2016).

This study is the first to construct a nomogram that combines both patient characteristics and clinical interventions for predicting the clinical outcome of patients presenting with AUGIB at an ED. The nomogram shows a good discriminative ability. However, this study has several limitations. First, this was a single-center retrospective study, and therefore its clinical usefulness remains to be assessed through external validation. Second, we only used the first available data obtained at the time of emergency admission for each patient, and therefore did not consider any subsequent changes. Thus, further research is required to validate this model. Third, not all patients underwent endoscopy and the diagnosis of UGIB was mainly based on symptoms, signs, and laboratory tests. Although patients’ electronic medical records were carefully reviewed, it is possible that the exact site of the bleeding of some patients may not be able to be identified. Moreover, we did not collect creatinine clearance and electrocardiogram, which maybe associated with blood potassium concentration. Therefore, we could not determine the correlation between these factors and death. However, we built a model with good performance. The addition of other variables would not significantly improve the prediction effect of the model. Finally, the outcome in our study was emergency mortality risk. Whether this model is applicable to predict in-hospital death, needs further research.

## Conclusions

We established and internally validated a nomogram to predict mortality among patients with AUGIB presenting at an ED. The nomogram was based on five readily available clinical predictors and details of clinical interventions. This model may be of value for individual risk assessments and for guiding staff in selecting interventions that are most likely to improve patient outcomes.

## Supplemental Information

10.7717/peerj.11656/supp-1 Supplemental Information 1 Raw data.

10.7717/peerj.11656/supp-2 Supplemental Information 2 Receiver operating characteristic curve of training group and validating group.

10.7717/peerj.11656/supp-3 Supplemental Information 3 Receiver operating characteristic curves, internal validation and decision curve analysis of the nomogram in subgroups undergoing and not undergoing endoscopy. (A) The area under the curve, (B) internal validation model, and (C) decision curve analysis for patients with endoscopy. (D) The area under the curve, (E) internal validation model, and (F) decision curve analysis for patients without endoscopy.

10.7717/peerj.11656/supp-4 Supplemental Information 4 Reclassification analyses for nomogram compared with different prognostic models for death in ED or death within 24 h after leaving the ED in patients with AUGIB, utilizing the continuous version of the net reclassification improvement approach.

## Additional Information and Declarations

### Data Availability

The following information was supplied regarding data availability: The raw measurements are available in the Supplemental File.
