# Supplementary figures and images for: Prediction model of emergency mortality risk in patients with acute upper gastrointestinal bleeding: a retrospective study
Source: PeerJ. 2021 Jun 24;9:e11656. doi: 10.7717/peerj.11656 (PMC8236237; doi:10.7717/peerj.11656)

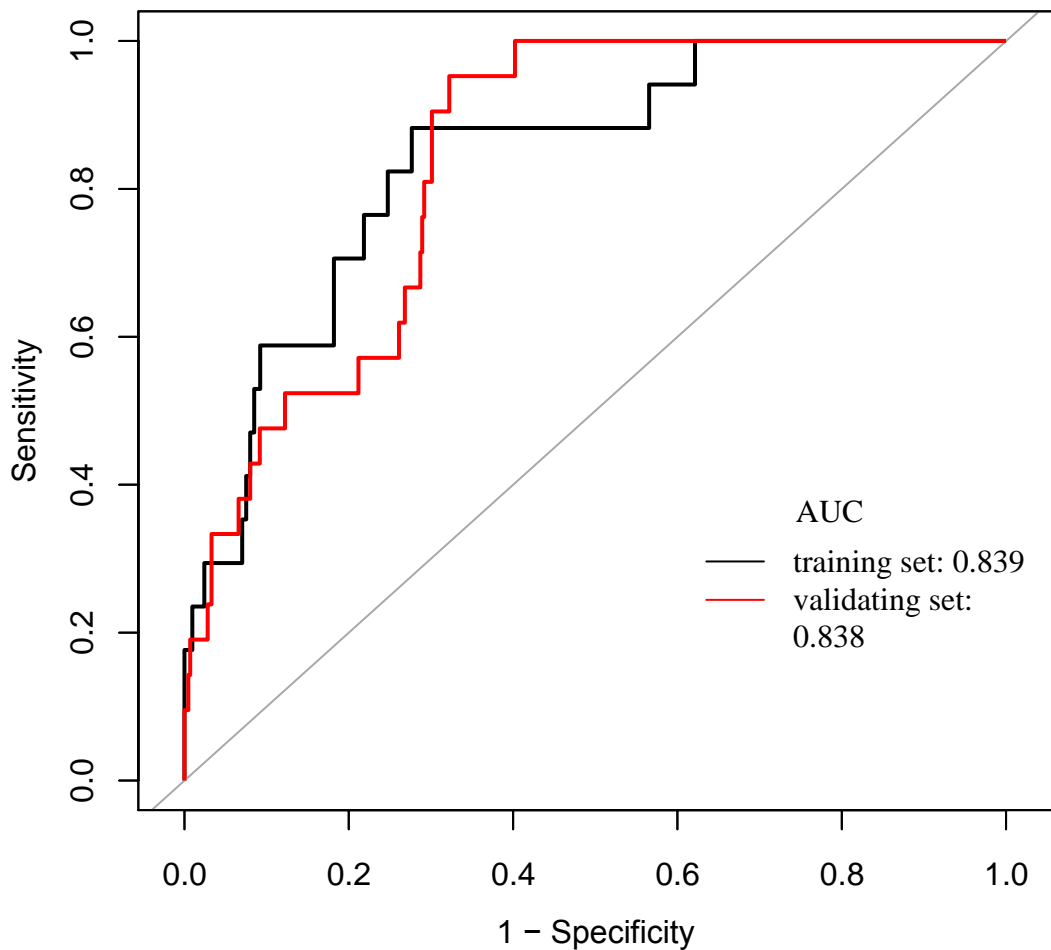

Supplement: Supplemental Information 2 [file peerj-09-11656-s002.pdf]

A

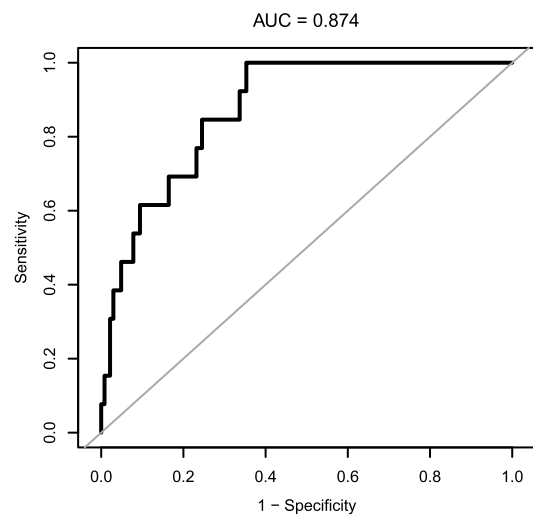

B

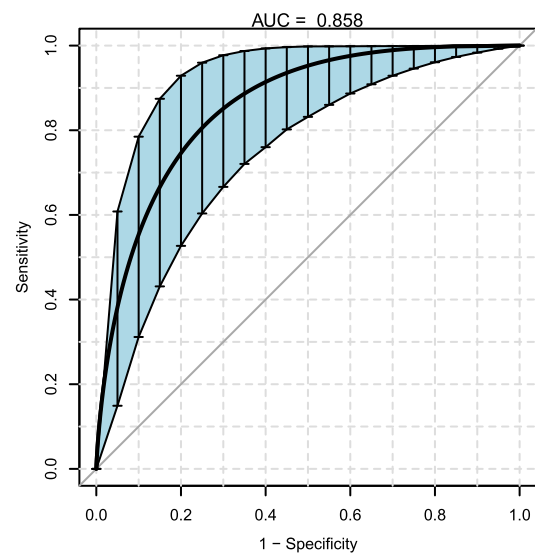

C

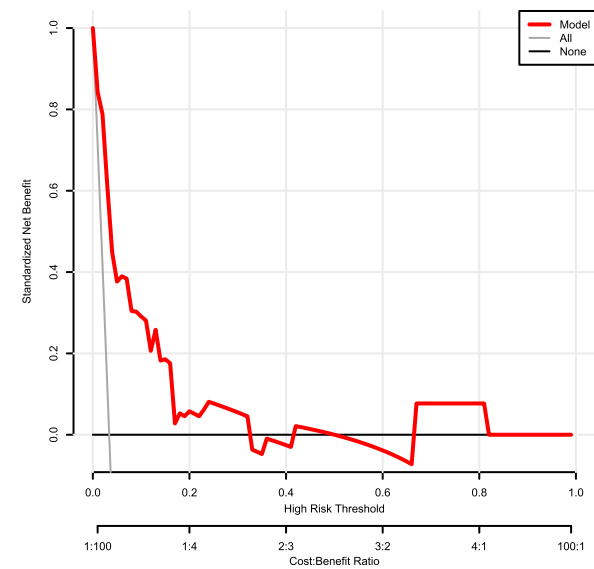

D

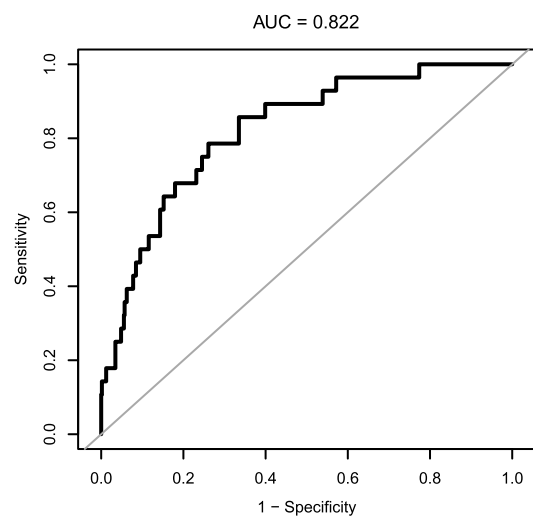

E

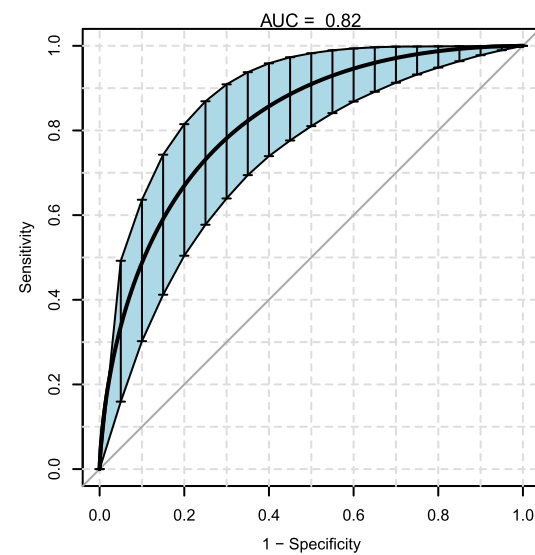

F

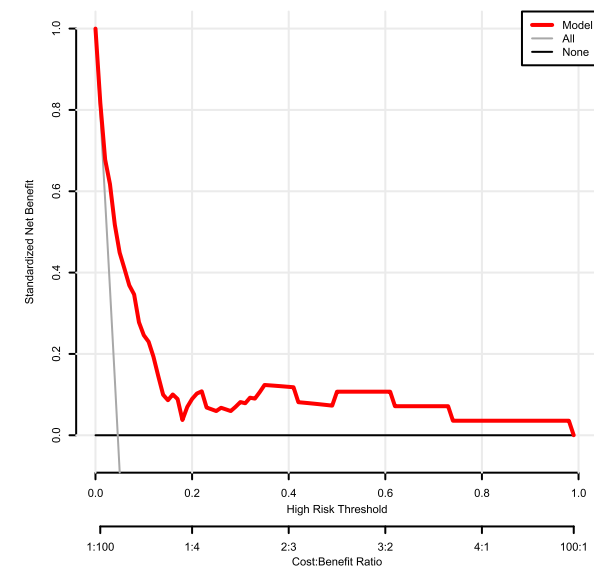

Supplement: Supplemental Information 3 — (A) The area under the curve, (B) internal validation model, and (C) decision curve analysis for patients with endoscopy. (D) The area under the curve, (E) internal validation model, and (F) decision curve analysis for patients without endoscopy. [file peerj-09-11656-s003.pdf]
