# Supplementary material for: Prediction model of emergency mortality risk in patients with acute upper gastrointestinal bleeding: a retrospective study
Source: PeerJ. 2021 Jun 24;9:e11656. doi: 10.7717/peerj.11656 (PMC8236237; doi:10.7717/peerj.11656)
Supplement: Supplemental Information 4 [file peerj-09-11656-s004.doc]

|  | NRI | P |
| --- | --- | --- |
| Compared with MGBS |  |  |
| Among event subjects | 39.47% | <0.001 |
| Among non-event subjects | -7.6% | <0.001 |
| Overall (95% CI) | 31.87% (48.12-15.63) | 0.001 |
| Compared with GBS |  |  |
| Among event subjects | 31.58% | <0.001 |
| Among non-event subjects | -3.38% | 0.084 |
| Overall (95% CI) | 28.2% (43.66-12.74) | <0.001 |
| Compared with PERS |  |  |
| Among event subjects | 18.42% | 0.025 |
| Among non-event subjects | 12.31% | <0.001 |
| Overall (95% CI) | 30.73% (47.58-13.88) | <0.001 |
| Compared with AIMS65 |  |  |
| Among event subjects | 5.56% | 0.410 |
| Among non-event subjects | 39.11% | <0.001 |
| Overall (95% CI) | 44.66% (58.70-30.62) | <0.001 |

AUGIB, acute upper gastrointestinal bleeding; ED, emergency department; NRI, net reclassification improvement; GBS, Glasgow-Blatchford bleeding score; MGBS, modified Glasgow-Blatchford bleeding score; PERS, Pre-Endoscopic Rockall Score; CI, confidence interval;
